# (4-Fluoro­phen­yl)(1*H*-pyrrol-2-yl)methan­one

**DOI:** 10.1107/S1600536812025871

**Published:** 2012-06-13

**Authors:** V. Prakash, Kamini Kapoor, M. Shet Prakash, Vivek K. Gupta, Rajni Kant

**Affiliations:** a Shirdi Sai Engineering College, Anekal, Bangalore 562 106, India; b X-ray Crystallography Laboratory, Post-Graduate Department of Physics & Electronics, University of Jammu, Jammu Tawi 180 006, India; c Department of Chemistry, University College of Science, Tumkur University, Tumkur, India; d Centre for Advanced Materials, Tumkur University, Tumkur, India

## Abstract

In the title mol­ecule, C_11_H_8_FNO, the dihedral angle between the pyrrole and benzene rings is 49.16 (6)°. In the crystal, adjacent mol­ecules are linked by pairs of N—H⋯O hydrogen bonds, forming inversion dimers.

## Related literature

For background to pyrrole derivatives and their applications, see: Fischer & Orth (1934 ▶); Mohamed *et al.* (2009 ▶). For related structures, see: English *et al.* (1980 ▶). For bond-length data, see: Allen *et al.* (1987 ▶). 
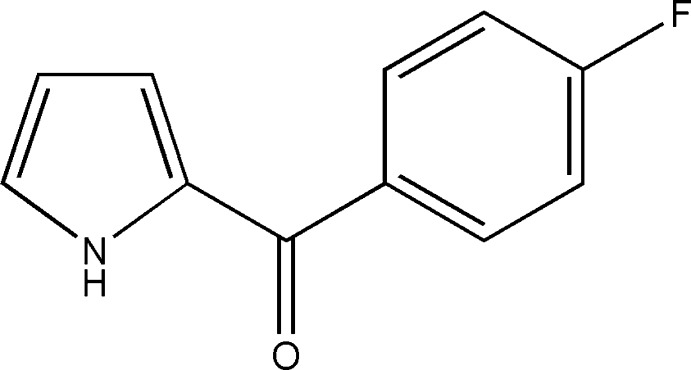


## Experimental

### 

#### Crystal data

C_11_H_8_FNO
*M* *_r_* = 189.18
Triclinic, 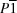

*a* = 3.8957 (2) Å
*b* = 10.7053 (5) Å
*c* = 11.1421 (6) Å
α = 99.167 (4)°
β = 95.951 (4)°
γ = 98.699 (4)°
*V* = 449.56 (4) Å^3^
*Z* = 2
Mo *K*α radiation
μ = 0.11 mm^−1^
*T* = 293 K
0.3 × 0.2 × 0.1 mm

#### Data collection

Oxford Diffraction Xcalibur Sapphire3 diffractometer
Absorption correction: multi-scan (*CrysAlis PRO*; Oxford Diffraction, 2010 ▶) *T*_min_ = 0.980, *T*_max_ = 1.000
10652 measured reflections
1762 independent reflections
1410 reflections with *I* > 2σ(*I*)
*R*_int_ = 0.027

#### Refinement

*R*[*F* ^2^ > 2σ(*F* ^2^)] = 0.038
*wR*(*F* ^2^) = 0.098
*S* = 1.03
1762 reflections
128 parameters
H-atom parameters constrained
Δρ_max_ = 0.16 e Å^−3^
Δρ_min_ = −0.15 e Å^−3^

### 

Data collection: *CrysAlis PRO* (Oxford Diffraction,2010 ▶); cell refinement: *CrysAlis PRO*; data reduction: *CrysAlis PRO*; program(s) used to solve structure: *SHELXS97* (Sheldrick, 2008 ▶); program(s) used to refine structure: *SHELXL97* (Sheldrick, 2008 ▶); molecular graphics: *ORTEP-3 for Windows* (Farrugia, 1997 ▶); software used to prepare material for publication: *PLATON* (Spek, 2009 ▶).

## Supplementary Material

Crystal structure: contains datablock(s) I, global. DOI: 10.1107/S1600536812025871/gk2499sup1.cif

Structure factors: contains datablock(s) I. DOI: 10.1107/S1600536812025871/gk2499Isup2.hkl

Supplementary material file. DOI: 10.1107/S1600536812025871/gk2499Isup3.cml

Additional supplementary materials:  crystallographic information; 3D view; checkCIF report

## Figures and Tables

**Table 1:** Hydrogen-bond geometry (Å, °)

*D*—H⋯*A*	*D*—H	H⋯*A*	*D*⋯*A*	*D*—H⋯*A*
N1—H1⋯O6^i^	0.86	2.06	2.865 (2)	157

Symmetry code: (i) 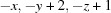.

**Fig. 1.**
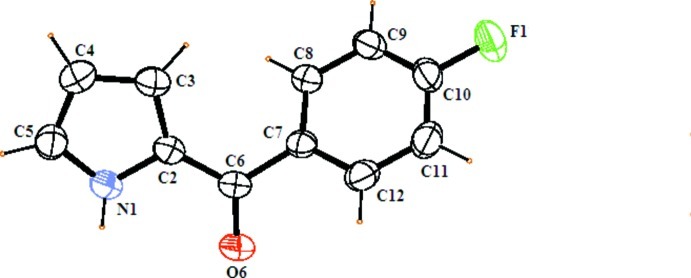
ORTEP view of the molecule with the atom-labeling scheme. The thermal ellipsoids are drawn at the 40% probability level. H atoms are shown as small spheres of arbitrary radii.

**Fig. 2.**
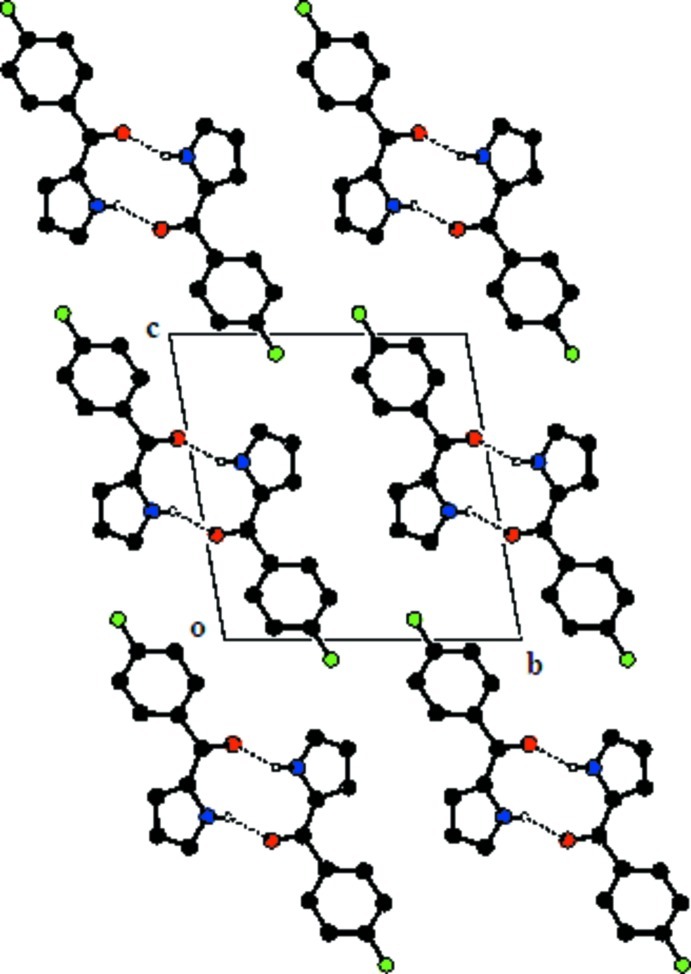
A molecular packing view of the title compound, showing intermolecular interactions. For clarity, hydrogen atoms which are not involved in hydrogen bonding have been omitted.

## supplementary crystallographic information

### Comment

The chemistry of pyrrole compounds and biological activities of the related compounds have been extensively studied (Fischer & Orth, 1934; Mohamed *et al.*, 2009). With the view of biological importance, the title compound was synthesized and its crystal structure is reported here.

Bond lengths and angles in the title compound (Fig. 1) have normal values (Allen *et al.*, 1987) and are comparable with the similar crystal structures solved earlier (English *et al.*, 1980). The pyrrole and benzene rings are planar with maximum deviations of 0.004 (2) Å and -0.009 (2) Å, respectively. The two rings are not coplanar with the dihedral angle being 49.16 (6)°. The crystal packing is stabilized by N—H···O intermolecular interactions, generating centrosymmetric dimers (Fig. 2).

### Experimental

Amide-phosphoryl complex was prepared by treating 1 equiv. of *N*,*N*-dimethyl-4-fluorobenzamide with 3 equiv. of POCl_3_ at room temperature and stirred for 6 h. The above complex was treated with pyrrole in anhydrous 1,2-dichloroethane at 25°C and stirred for one hour and kept overnight. The resulting mixture was hydrolyzed using saturated sodium carbonate solution, followed by heating for 45 minutes to obtain the title compound. The title compound was extracted using 1,2-dichloroethane. Single crystals required for X-ray diffraction were obtained by slow evaporation of the methanolic solution of the compound.

### Refinement

All H atoms were positioned geometrically and were treated as riding on their parent atoms, with d(N—H)= 0.86 Å and d(C—H) = 0.93 Å for aromatic, and with *U*_iso_(H) = 1.2*U*_eq_(C_aryl_, N).

### Crystal data

**Table:**

C_11_H_8_FNO	*Z* = 2
*M**_r_* = 189.18	*F*(000) = 196
Triclinic, *P*1	*D*_x_ = 1.398 Mg m^−^^3^
Hall symbol: -P 1	Mo *K*α radiation, λ = 0.71073 Å
*a* = 3.8957 (2) Å	Cell parameters from 4740 reflections
*b* = 10.7053 (5) Å	θ = 3.7–28.9°
*c* = 11.1421 (6) Å	µ = 0.11 mm^−^^1^
α = 99.167 (4)°	*T* = 293 K
β = 95.951 (4)°	Plate, white
γ = 98.699 (4)°	0.3 × 0.2 × 0.1 mm
*V* = 449.56 (4) Å^3^	

### Data collection

**Table:**

Oxford Diffraction Xcalibur Sapphire3 diffractometer	1762 independent reflections
Radiation source: fine-focus sealed tube	1410 reflections with *I* > 2σ(*I*)
Graphite monochromator	*R*_int_ = 0.027
Detector resolution: 16.1049 pixels mm^-1^	θ_max_ = 26.0°, θ_min_ = 3.7°
ω scan	*h* = −4→4
Absorption correction: multi-scan (*CrysAlis PRO*; Oxford Diffraction, 2010)	*k* = −13→13
*T*_min_ = 0.980, *T*_max_ = 1.000	*l* = −13→13
10652 measured reflections	

### Refinement

**Table:**

Refinement on *F*^2^	Secondary atom site location: difference Fourier map
Least-squares matrix: full	Hydrogen site location: inferred from neighbouring sites
*R*[*F*^2^ > 2σ(*F*^2^)] = 0.038	H-atom parameters constrained
*wR*(*F*^2^) = 0.098	*w* = 1/[σ^2^(*F*_o_^2^) + (0.0393*P*)^2^ + 0.1079*P*] where *P* = (*F*_o_^2^ + 2*F*_c_^2^)/3
*S* = 1.03	(Δ/σ)_max_ = 0.001
1762 reflections	Δρ_max_ = 0.16 e Å^−^^3^
128 parameters	Δρ_min_ = −0.15 e Å^−^^3^
0 restraints	Extinction correction: *SHELXL*, Fc^*^=kFc[1+0.001xFc^2^λ^3^/sin(2θ)]^-1/4^
Primary atom site location: structure-invariant direct methods	Extinction coefficient: 0.068 (8)

### Special details

**Table:**

Experimental. *CrysAlis PRO*, Oxford Diffraction Ltd., Version 1.171.34.40 (release 27–08-2010 CrysAlis171. NET) (compiled Aug 27 2010,11:50:40) Empirical absorption correction using spherical harmonics, implemented in SCALE3 ABSPACK scaling algorithm.
Geometry. All e.s.d.'s (except the e.s.d. in the dihedral angle between two l.s. planes) are estimated using the full covariance matrix. The cell e.s.d.'s are taken into account individually in the estimation of e.s.d.'s in distances, angles and torsion angles; correlations between e.s.d.'s in cell parameters are only used when they are defined by crystal symmetry. An approximate (isotropic) treatment of cell e.s.d.'s is used for estimating e.s.d.'s involving l.s. planes.
Refinement. Refinement of *F*^2^ against ALL reflections. The weighted *R*-factor *wR* and goodness of fit *S* are based on *F*^2^, conventional *R*-factors *R* are based on *F*, with *F* set to zero for negative *F*^2^. The threshold expression of *F*^2^ > σ(*F*^2^) is used only for calculating *R*-factors(gt) *etc*. and is not relevant to the choice of reflections for refinement. *R*-factors based on *F*^2^ are statistically about twice as large as those based on *F*, and *R*- factors based on ALL data will be even larger.

### Fractional atomic coordinates and isotropic or equivalent isotropic displacement parameters (Å^2^)

**Table:**

	*x*	*y*	*z*	*U*_iso_*/*U*_eq_	
N1	0.1668 (3)	0.83359 (12)	0.41956 (11)	0.0485 (3)	
H1	0.0862	0.9039	0.4184	0.058*	
C2	0.2594 (4)	0.78614 (13)	0.52317 (13)	0.0415 (3)	
C3	0.3794 (4)	0.67263 (14)	0.48471 (15)	0.0474 (4)	
H3	0.4618	0.6192	0.5350	0.057*	
C4	0.3545 (4)	0.65312 (15)	0.35771 (15)	0.0538 (4)	
H4	0.4169	0.5845	0.3074	0.065*	
C5	0.2207 (4)	0.75399 (16)	0.32037 (14)	0.0543 (4)	
H5	0.1750	0.7653	0.2396	0.065*	
C6	0.2390 (4)	0.85622 (13)	0.64280 (14)	0.0428 (4)	
O6	0.1574 (3)	0.96424 (10)	0.65746 (10)	0.0600 (4)	
C7	0.3239 (4)	0.79769 (13)	0.75287 (13)	0.0414 (3)	
C8	0.1941 (4)	0.67098 (14)	0.75797 (14)	0.0468 (4)	
H8	0.0594	0.6185	0.6895	0.056*	
C9	0.2626 (5)	0.62175 (16)	0.86362 (15)	0.0554 (4)	
H9	0.1716	0.5373	0.8678	0.066*	
C10	0.4675 (5)	0.70044 (18)	0.96151 (15)	0.0597 (5)	
C11	0.6022 (5)	0.82580 (18)	0.96050 (15)	0.0619 (5)	
H11	0.7410	0.8768	1.0290	0.074*	
C12	0.5269 (4)	0.87470 (15)	0.85531 (15)	0.0525 (4)	
H12	0.6130	0.9601	0.8531	0.063*	
F1	0.5388 (4)	0.65272 (12)	1.06567 (9)	0.0951 (5)	

### Atomic displacement parameters (Å^2^)

**Table:**

	*U*^11^	*U*^22^	*U*^33^	*U*^12^	*U*^13^	*U*^23^
N1	0.0574 (8)	0.0403 (7)	0.0514 (8)	0.0120 (6)	0.0089 (6)	0.0141 (6)
C2	0.0402 (8)	0.0380 (8)	0.0473 (8)	0.0057 (6)	0.0047 (6)	0.0117 (6)
C3	0.0459 (8)	0.0416 (8)	0.0565 (9)	0.0120 (6)	0.0054 (7)	0.0106 (7)
C4	0.0548 (10)	0.0477 (9)	0.0577 (10)	0.0109 (7)	0.0114 (7)	0.0012 (7)
C5	0.0608 (10)	0.0544 (10)	0.0464 (9)	0.0065 (8)	0.0083 (7)	0.0077 (7)
C6	0.0426 (8)	0.0345 (7)	0.0526 (9)	0.0077 (6)	0.0069 (6)	0.0104 (6)
O6	0.0852 (9)	0.0397 (6)	0.0603 (7)	0.0228 (6)	0.0102 (6)	0.0122 (5)
C7	0.0420 (8)	0.0390 (8)	0.0453 (8)	0.0143 (6)	0.0065 (6)	0.0062 (6)
C8	0.0525 (9)	0.0410 (8)	0.0477 (9)	0.0120 (7)	0.0031 (7)	0.0087 (6)
C9	0.0711 (11)	0.0479 (9)	0.0543 (10)	0.0216 (8)	0.0121 (8)	0.0169 (8)
C10	0.0792 (12)	0.0679 (11)	0.0415 (9)	0.0392 (9)	0.0080 (8)	0.0131 (8)
C11	0.0695 (11)	0.0666 (12)	0.0460 (9)	0.0260 (9)	−0.0043 (8)	−0.0063 (8)
C12	0.0573 (10)	0.0433 (9)	0.0547 (10)	0.0129 (7)	0.0055 (7)	−0.0005 (7)
F1	0.1500 (12)	0.1005 (9)	0.0478 (6)	0.0585 (8)	0.0025 (7)	0.0240 (6)

### Geometric parameters (Å, º)

**Table:**

N1—C5	1.339 (2)	C7—C8	1.386 (2)
N1—C2	1.3699 (18)	C7—C12	1.387 (2)
N1—H1	0.8600	C8—C9	1.382 (2)
C2—C3	1.388 (2)	C8—H8	0.9300
C2—C6	1.438 (2)	C9—C10	1.364 (3)
C3—C4	1.388 (2)	C9—H9	0.9300
C3—H3	0.9300	C10—F1	1.3602 (18)
C4—C5	1.371 (2)	C10—C11	1.367 (3)
C4—H4	0.9300	C11—C12	1.380 (2)
C5—H5	0.9300	C11—H11	0.9300
C6—O6	1.2357 (17)	C12—H12	0.9300
C6—C7	1.493 (2)		
			
C5—N1—C2	109.79 (12)	C8—C7—C12	119.05 (14)
C5—N1—H1	125.1	C8—C7—C6	122.28 (13)
C2—N1—H1	125.1	C12—C7—C6	118.61 (13)
N1—C2—C3	106.57 (13)	C9—C8—C7	120.79 (15)
N1—C2—C6	120.91 (12)	C9—C8—H8	119.6
C3—C2—C6	132.43 (13)	C7—C8—H8	119.6
C2—C3—C4	107.76 (13)	C10—C9—C8	118.13 (15)
C2—C3—H3	126.1	C10—C9—H9	120.9
C4—C3—H3	126.1	C8—C9—H9	120.9
C5—C4—C3	107.18 (14)	F1—C10—C9	118.64 (17)
C5—C4—H4	126.4	F1—C10—C11	118.26 (16)
C3—C4—H4	126.4	C9—C10—C11	123.10 (15)
N1—C5—C4	108.69 (14)	C10—C11—C12	118.31 (15)
N1—C5—H5	125.7	C10—C11—H11	120.8
C4—C5—H5	125.7	C12—C11—H11	120.8
O6—C6—C2	121.97 (13)	C11—C12—C7	120.61 (15)
O6—C6—C7	118.92 (13)	C11—C12—H12	119.7
C2—C6—C7	119.11 (12)	C7—C12—H12	119.7
			
C5—N1—C2—C3	0.71 (17)	O6—C6—C7—C12	−42.0 (2)
C5—N1—C2—C6	177.63 (14)	C2—C6—C7—C12	137.16 (15)
N1—C2—C3—C4	−0.42 (17)	C12—C7—C8—C9	0.5 (2)
C6—C2—C3—C4	−176.84 (15)	C6—C7—C8—C9	−176.53 (14)
C2—C3—C4—C5	−0.01 (18)	C7—C8—C9—C10	−1.4 (2)
C2—N1—C5—C4	−0.73 (18)	C8—C9—C10—F1	−179.51 (14)
C3—C4—C5—N1	0.45 (19)	C8—C9—C10—C11	1.2 (3)
N1—C2—C6—O6	−4.2 (2)	F1—C10—C11—C12	−179.32 (15)
C3—C2—C6—O6	171.82 (16)	C9—C10—C11—C12	0.0 (3)
N1—C2—C6—C7	176.72 (13)	C10—C11—C12—C7	−0.9 (2)
C3—C2—C6—C7	−7.3 (2)	C8—C7—C12—C11	0.7 (2)
O6—C6—C7—C8	135.08 (16)	C6—C7—C12—C11	177.84 (14)
C2—C6—C7—C8	−45.8 (2)		

### Hydrogen-bond geometry (Å, º)

**Table:**

*D*—H···*A*	*D*—H	H···*A*	*D*···*A*	*D*—H···*A*
N1—H1···O6^i^	0.86	2.06	2.865 (2)	157

Symmetry code: (i) −*x*, −*y*+2, −*z*+1.
